# Smoking Behavior among Adolescents: The Lebanese Experience with Cigarette Smoking and Waterpipe Use

**DOI:** 10.3390/ijerph19095679

**Published:** 2022-05-06

**Authors:** Marwan Akel, Fouad Sakr, Iqbal Fahs, Ahmad Dimassi, Mariam Dabbous, Virginie Ehlinger, Pascale Salameh, Emmanuelle Godeau

**Affiliations:** 1 School of Pharmacy, Lebanese International University, Beirut 1103, Lebanon; marwan.akel@liu.edu.lb (M.A.); iqbal.fahs@liu.edu.lb (I.F.); ahmad.dimassi@liu.edu.lb (A.D.); mariam.dabbous@liu.edu.lb (M.D.); 2 INSPECT-LB (Institut National de Santé Publique, d’Épidémiologie Clinique et de Toxicologie-Liban), Beirut 6573, Lebanon; pascalesalameh1@hotmail.com; 3 International Pharmaceutical Federation (FIP), 2517 The Hague, The Netherlands; 4 UMR 1295 CERPOP (Centre for Epidemiology and Research in Population Health), INSERM, Toulouse University III Paul Sabatier, Team SPHERE, 31059 Toulouse, France; virginie.ehlinger@inserm.fr (V.E.); emmanuelle.godeau@ehesp.fr (E.G.); 5 INSERM U955, Institut Mondor de Recherche Biomédicale, Université Paris-Est Créteil, 94000 Créteil, France; 6 School of Education, Lebanese International University, Beirut 1103, Lebanon; 7 Faculty of Public Health, Lebanese University, Beirut 6573, Lebanon; 8 Department of Primary Care and Population Health, University of Nicosia Medical School, Nicosia 2408, Cyprus; 9 Ecole des Hautes Etudes en Sante Publique, 35043 Rennes, France

**Keywords:** smoking, children, adolescents, school aged, cigarette, waterpipe

## Abstract

(1) Background: The study aims to assess cigarette smoking and waterpipe experimentation among Lebanese adolescent school students with respect to their gender, region, age, and socioeconomic status. (2) Methods: This is a cross-sectional study, where students between 11 to 18 years of age were included from all over Lebanon. (3) Results: A total of 1133 students were interviewed. The total proportion of adolescents who ever experimented with cigarette smoking was 24.5%. Males experimented with cigarette smoking more commonly than females (31.9% vs. 19.1%; *p* < 0.001). Cigarette smoking experimentation was higher among students from the Beirut area (33.6%; *p* < 0.001) in comparison to other regions, and among those with poor health perception (29.1% vs. 19.8%; *p* < 0.001) compared to students with excellent health perception. The total proportion of adolescents who ever used a waterpipe was 33.9%. Waterpipes were significantly more experimented with among males than females (40.3% vs. 29.8%; *p* < 0.001), and among students with bad perception about their health (39.4% vs. 28.9%; *p* < 0.001). Adolescents who experimented with both cigarettes and waterpipes constitute 22.2% of the studied sample. (4) Conclusions: The rate of tobacco product use is alarming and constitutes a major public health issue for adolescents that urgently needs intervention. The findings raise important policy implications for the development of cigarette smoking prevention programs for youth.

## 1. Introduction

Smoking is a major cause of global morbidity and mortality [1]. It harms nearly every organ of the body and reduces the overall health of smokers. Worldwide, tobacco use causes more than 7 million deaths per year [2]. Cigarette smoking causes more than 480,000 deaths every year in the United States [3]. Lebanon is ranked third in the world for highest cigarette consumption per capita with 3500 annual deaths because of tobacco-related diseases [4]. The global annual deaths related to smoking are expected to reach 10 million by the year 2025, with 7 million deaths occurring in the developing world [5]. 

Adolescence is a critical age in one’s life. Its practices are most likely to have an impact that would last until adulthood, particularly those picked up in school. Despite the fact that the majority of smoking-related deaths occur in middle-aged and elderly people, smoking behavior is undeniably established in adolescence [6,7]. Globally, tobacco use among adolescents is increasing, even if those rates are clearly declining in western countries [7,8]. The estimated numbers of boys and girls aged 13–15 years old who smoke cigarettes or use smokeless tobacco products are approximately 25 million and 13 million, respectively [9]. Moreover, the prevalence of cigarette smoking among youth in the US is estimated to be one in four high school students [7]. People who start smoking at a younger age have a higher risk of becoming daily smokers [10]. Those who consume tobacco products at an early age are more likely to have a longer duration of tobacco use than those who start later in life [9]. 

Although the waterpipe was originally an eastern smoking device, evidence suggests that it had made its way to the cafes of the United States of America and several western and eastern European countries [11,12]. The use of the waterpipe is increasing worldwide, mainly among youth and young adults, where it is becoming a new global public health threat [13]. The waterpipe is becoming the first tobacco product tried by youth [14]. Several studies have shown that cigarette-naïve youth who smoke waterpipes are at a higher risk of initiating cigarette smoking [15,16]. Waterpipe consumption is linked to diseases similarly as cigarette consumption, like lung cancer, oral cancer, cardiovascular disease, and respiratory disease [17,18]. Nonetheless, people consider waterpipe tobacco to be less harmful than cigarette tobacco mainly due to the socialization, relaxation and entertainment that waterpipe tobacco use brings to the user [19]. 

While the problem of cigarette smoking among adolescent school students has been highly addressed in developed countries, only a few similar studies have been conducted in developing countries, in particular, the Arab ones. Information on tobacco use among adolescents is limited in Lebanon. In a study on patterns of tobacco use, results from the 2005 Global Youth Tobacco Survey in Lebanon have revealed that almost 3 in 10 students reported that they had ever smoked a cigarette [20]. The majority of students reported using some form of tobacco, with 10% smoking cigarettes and more than 55% using some other forms of tobacco like waterpipes, pipes and cigars. Use of other tobacco products was significantly higher than cigarette smoking for both boys and girls [20]. In a study done in the Eastern Mediterranean Region and among adolescents aged 13–15 years old, waterpipe prevalence in the past 30-days was highest in Lebanon (36.9%) and the West Bank (32.7%) [12]. The World Health Organization Global Action Plan is aiming toward a 25% relative reduction in tobacco use by 2025, though the evidence suggests that cigarette smoking and waterpipe use may compromise this target given their anecdotal increase in prevalence across multiple settings [21]. 

With such alarming percentages regarding cigarette and waterpipe smoking among adolescents, data about the national prevalence of tobacco use and smoking trends are urgently demanded to make decisions about future smoking prevention policies. Hence, this study sought to assess cigarette smoking and waterpipe experimentation among adolescent school students in Lebanon with respect to their gender, region, age, and socioeconomic status. 

## 2. Materials and Methods

### 2.1. Study Design and Setting

This was a prospective cross-sectional observational study, where different schools across Lebanon were approached for inclusion. The study was conducted over the course of seven months, from October 2016 until April 2017. The target population was Lebanese adolescent students of private and public schools, using a convenient sampling method. Out of 61 schools approached, only 32 accepted to participate in the study. The included schools were distributed over four Lebanese districts (Beirut, Mount Lebanon, Bekaa and North). 

### 2.2. Ethical Aspect

The study was approved by the institutional review boards of the targeted schools. A proposal was sent to the schools prior to study enrollment, and the study did not pose any risk to the participants. Parents and students were informed and could refuse to participate, though collected data were stripped of any personal identified information. 

### 2.3. Data Collection

Interviews were performed on site by the principal investigator without interference or bias using a data collection sheet. The questionnaire was required to be completed by each student individually. Each section of the questionnaire dealt with a specific topic, including demographics, lifestyle, familial relationships, tobacco use, socio-economic status, and health perception. The questionnaire comprised a majority of closed-ended questions. The first step in data collection was to gain the schools’ administrations’ approvals to engage their students in the study, for which proposal letters were composed and delivered. The letter included a detailed description of the purpose of the study, the target population, the questionnaire and how it should be completed and why their school was chosen. Once the approval was received, the data collection proceeded with the questionnaires being distributed to the students in order to be completed. A pilot study was performed prior to proceeding with the data collection, during which the estimated completion time of the questionnaire was around 35 min. The students were carefully instructed on how to complete the questionnaire, and they were granted the right to skip any question they considered relatively inappropriate or uncomfortable. In addition, they were instructed to answer as quickly and honestly as possible. Students were instructed not to write their first or family names in order to maintain the questionnaire’s anonymity and the study’s integrity. Each student took his or her time individually in order to complete the sixty-one questions of the questionnaire, after which all questionnaires were collected. 

### 2.4. Measures

- Cigarette and waterpipe status were defined on the basis of the question “How often do you smoke tobacco at present?” Possible responses included “every day”, “at least once a week, but not every day”, “less than once a week”, or “never”. Adolescents who smoked “less than once a week” or more often were considered to be cigarette smokers. The age of starting smoking was based on the question “At which age did you smoke the first cigarette?” [22].
- The Self-rated Health Measure (SRH) was used to assess perceived health status. This measure includes four response categories (excellent; good; fair; or poor) in response to the question “would you say your health is...?” [22].
- The Family Affluence Scale (FAS) was used as an indicator of socio-economic status. FAS is a four-item measure of family wealth which has been developed in the WHO Health Behavior in School-aged Children Study. The Health Behavior in School-Aged Children study (HBSC) uses the FAS as a tool to identify the socioeconomic status of children and adolescents and seems to be a valid instrument to measure adolescents’ socioeconomic status [23,24]. It comprises four items, including parental car ownership (“Does your family own a car, van, or a truck?” (0, 1, 2)), sharing or not sharing bedroom (“Do you have your own room?” (1, 0)), number of holidays per year (“During the past 12 months, how many times did you travel away on holiday with your family?” (0, 1, 2, 3)), having computers at home (“How many computers does your family own?” (0, 1, 2, 3)) [22]. The composite FAS score was calculated for each adolescent by adding of the four items (ranging from 0–9) and further categorisation into low (0–5), medium (6–7) and high (8–9) [25].

### 2.5. Inclusion and Exclusion Criteria

The students whose ages ranged between 11 to 18 years old were considered eligible for enrollment. Students less than 11 years or more than 18 years of age, students attending schools outside the previously mentioned Lebanese regions, children not enrolled in schools and students of non-Lebanese nationality were excluded.

### 2.6. Outcomes

The primary outcome describes the Lebanese youth’s consumption of cigarettes and waterpipes across the country in terms of starting age, frequency of use, and gender and regional distributions. The secondary aim assesses factors that may be linked or play a role in such behaviors including age, gender, socio-economic status and health perception. 

### 2.7. Statistical Analysis

STATA Software was used for statistical analysis. Descriptive statistics were adopted to analyze the collected data. Means and standard deviations (SD) were used for continuous variables such as the number of cigarettes smoked per week and the number of waterpipes smoked per week, while frequency tables were established for the categorical variables. Chi-square two by two testing was performed to reveal the significance between smoking and other variables such as gender, region, and socio-economic status. All reported *p*-values were two-sided, with the alpha set at a significance of 0.05. 

## 3. Results

### 3.1. Demographic Characteristics

A total of 1133 students were interviewed, out of which only 1117 were included in statistical analysis. Sixteen students were excluded due to the absence of data related to age, gender and socio-economic status. The majority of the students (68.3%) were from Mount Lebanon. The mean age of the sample was almost 15 years of age with no differences between males (15.32 ± 1.96) and females (15.17 ± 2.03). The highest proportion of students were 17 years and older (28.4 %). The studied sample was almost equally divided between males (45.5%) and females (54.4%). Those who perceived their heath as excellent were highest in Bekaa (53.1%), followed by Mount Lebanon (43.1%). Almost half of the enrolled students (49.9%) had a moderate FAS, reflecting a moderate socio-economic level. The proportion of students with high FAS was highest in Bekaa and lowest in North Lebanon. Table 1 illustrates the demographic characteristics of the studied participants. 

### 3.2. Tobacco Experimentation Based on Age and Region

**Figure 1:**
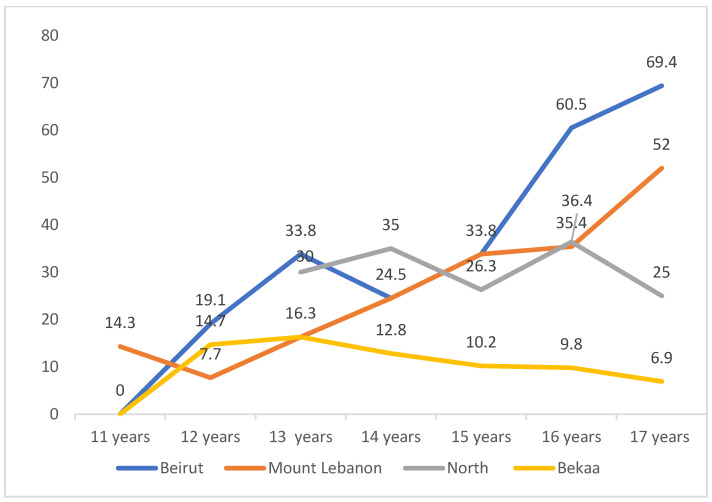
Proportion of boys who have ever experienced smoking according to age and region.

Tobacco experimentation was null among boys at the age of 11 years in Beirut. It started to increase gradually to reach a dramatic peak (60.5%) at age 16, and further increased at the age of 17 (69.4%). In Mount Lebanon, even before the age of 11 years, almost 15% of students experimented tobacco. This level increased to reach a peak at the age of 17 (52%) that is still lower than that of Beirut. In North Lebanon, tobacco experimentation started later at 13 years of age, with around 30% of students reporting use at this age. Such use in North Lebanon increased and decreased among the years, with a lower peak (36.4%) at age 16 years compared to Beirut. Tobacco use and experimentation among boys in Bekaa was almost the lowest at any age interval compared to other districts, with the highest experimentation of tobacco products at 13 years of age (16.3%). Figure 1 demonstrates the proportion of boys who have ever experienced smoking at different age intervals and among the four districts.

**Figure 2:**
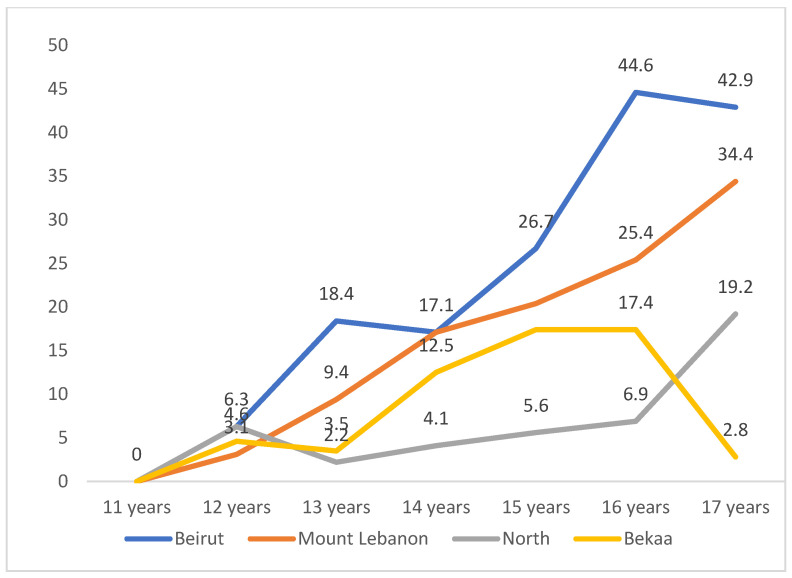
Proportion of girls who have ever experienced smoking according to age and region.

Tobacco experimentation among girls was lower compared to boys in almost all regions. The experimentation of tobacco increased slightly at the age of 12 years in all areas (3.1–3.6%) and continued to rise gradually to reach peak values at the age of 16 in Beirut (44.6%), Mount Lebanon (25.4%), Bekaa (17.4%) and North (6.9%). The peaks were lower than those reported by boys at this age and were highest in Beirut, followed by Mount Lebanon. Figure 2 demonstrates the proportion of girls who have ever experienced smoking at different age intervals and among the four districts.

### 3.3. Cigarette Smoking Modalities According to Gender, Region, FAS, and Health Perception 

The total proportion of adolescents who have ever experimented with cigarette smoking alone was 24.5%. Males experimented with cigarette smoking more commonly than females (31.9% vs. 19.1%; *p* < 0.001). Those who experimented with smoking were mainly from Beirut (33.6%; *p* < 0.001) with poor health perception (29.1% vs. 19.8%; *p* < 0.001). The frequency of daily smoking was much higher among males (7.3% vs. 3.0%; *p* < 0.001), in the Beirut area compared to other regions (16.6%; *p* < 0.001), among those with higher FAS (6.2%; *p* < 0.001), and among those with bad health perception (5.9% vs. 3.4%; *p* < 0.001). 

Daily consumption of <2 or >2 packs/day was significantly higher among boys (9.0% vs. 4.2%; *p* < 0.001), in the Beirut region, and among students with high FAS and poor health perception. Males started smoking at a significantly lower age compared to females and at any age thereafter. At an age of 11 years, 5.4% of males started smoking compared to 1.6% of female students (*p* < 0.001). Those in Mount Lebanon started smoking at an earlier age compared to Beirut students (4.2% vs. 2.2%; *p* < 0.001). Mainly those with high FAS started smoking at younger age. At the age of 11 years, smoking rates were higher among students with high FAS than those with moderate (4.1% vs. 3.5%; *p* = 0.004). Table 2 illustrates cigarette smoking modalities according to gender, region, FAS and health perception.

### 3.4. Waterpipe (WP) Modalities According to Gender, Region, FAS, and Health Perception

The total proportion of adolescents who ever used WP alone was 33.9%. WP was significantly more experimented among males (40.3% vs. 29.8%; *p* < 0.001), in the Beirut region (39.5%, *p* < 0.001), and among those with bad perception about their health (39.4% vs. 28.9%; *p* < 0.001). WP sessions/week were significantly higher among males (*p* < 0.001). The highest number of sessions (>2/week) was reported in Beirut. As the number of sessions increased, health perception was lower (*p* < 0.001). 

At the age of 11 years, males utilized WP more significantly than females (6.0% vs. 2.2%; *p* < 0.001). At higher age intervals, the difference between males and females regarding WP starting age was almost null, with a slightly higher proportion of females starting WP than males at the age of 16 and more (4.7% vs. 4.2%). Starting WP at any age was significantly higher in Beirut and Mount Lebanon compared to the Bekaa and North areas. 

FAS did not correlate with WP experimentation or WP sessions per week, but was significantly associated with WP starting age. Those with higher FAS started WP at a younger age. At the age of 11 years, 5.6% of high FAS students vs. 3.8% of low-to-average FAS started WP (*p* < 0.001). At any age, those with bad health perception started smoking at younger ages (*p* < 0.001). Table 3 demonstrates WP smoking modalities according to gender, region, FAS and health perception.

### 3.5. Both Cigarette Smoking and Waterpipe 

Adolescents who experimented with both cigarettes and WP constitute 22.2% of the studied sample. Those who experimented with both were significantly higher among Beirut students (30.8%, *p* < 0.001), males (28.7 vs. 16.9; *p* < 0.001), students with average-to-high FAS and poor health perception (25.9% vs. 22.4%; *p* < 0.001). Table 4 illustrates smoking and WP experimentation among adolescents based on region, gender, FAS and perceived health. 

## 4. Discussion

Tobacco use continues to be the leading cause of preventable morbidity and mortality worldwide. Due to the adoption of successful policies and interventions, tobacco use has been on the decline over the past 30 years in most developed societies, especially among adults. Trends in global tobacco adolescent use showed that while cigarette smoking is either stable or declining, other forms of tobacco use were showing a rising trend, most notably waterpipe smoking among young adolescents [26].

In the current study, the prevalence of experimenting with cigarette smoking, waterpipe smoking and both among adolescents were 24.5%, 33.98% and 22.8%, respectively. In a study on the patterns of tobacco use from the 2005 Global Youth Tobacco Survey in Lebanon, 27% reported that they had ever smoked a cigarette, which is almost comparable to the proportion in our study (24.5%) [20]. In another study on tobacco smoking among Lebanese university students, the overall prevalence of smoking in the sample was 40%. More than 20% had smoked only waterpipes, 7.6% smoked only cigarettes and 11.3% smoked both cigarettes and waterpipes. 

The prevalence of cigarette and overall smoking in the current study is higher than the rates reported in similar studies in North America, where the prevalence rates of cigarette smoking reached 13.8% [27]. Our results also demonstrated higher smoking experimentation compared to the neighboring countries. A recent study from Syria showed a prevalence rate of 11% for cigarette smoking among high school students with a mean age of 16 years [28]. However, higher prevalence rates were documented in Yemen among secondary school students [29], and in Kuwait among young men aged less than 20 years of age [30]. The increase in youth smoking in Lebanon echoes, but out paces, the World Health Organization (WHO) statistics available for other countries in the Middle East. For instance, in Egypt, 7.5% of youth between 13 and 15 years of age smoked in 2009 [31]. This number increased to 10.1% in 2014 [31]. Additionally, in Iraq, 13.4% of adolescents had used tobacco in 2009 [32]. In 2014, the number increased slightly to 14.1% [32].

Waterpipe smoking is on the rise among youth and adolescents in Lebanon [11]. This can be attributed to the misconception of waterpipe smoking versus cigarette smoking. According to Schroder et al., waterpipe smoking is socially acceptable contrary to cigarette smoking [33]. Adolescents considered waterpipe smoking to be a good experience. Furthermore, peer pressure was cited to be an influencing factor for waterpipe smoking. The misconception of waterpipes being better than cigarettes has been common between Lebanese youth. Nakkash et al., reported that waterpipe smoking is even more dangerous than cigarette smoking [34]. Waterpipes contain a wide range of toxins and pollutants, including carbon monoxide, nicotine, arsenic, formaldehyde and others. In addition, waterpipe smoking can lead to many diseases like lung cancer and tuberculosis. However, there is a widespread availability of waterpipes at restaurants, coffee shops, and for home delivery in Lebanon, which has a significant role in the rising phenomenon of waterpipe smoking. In addition, waterpipes are available at cheap prices, as cheap as 5000 Lebanese Pounds (equivalent to 3.3 US Dollars). Moreover, improvements to waterpipes have been made, and they are now available in different flavors and with colorful apparatus designs, both serving as attractants to waterpipe smoking [34].

We have found that males were superior to females in experimenting with cigarette smoking, waterpipe smoking, and both. This could be related mainly to the relatively conservative social norms and attitudes towards smoking, where women may avoid smoking cigarettes in front of men as a matter of respect and social discouragement. A cross-sectional study was conducted in 2004 in the Greater Beirut region. In total, 2443 students belonging to intermediate and secondary divisions were interviewed, with an average age of 15 years [35]. The prevalence of waterpipe smoking was greater than that of cigarette smoking, where 23.3% and 29.2% of intermediate students and secondary students, respectively, smoked waterpipes only [35]. The results are comparable to our study. Only a minority smoked both cigarettes and waterpipes, with 6.3% for intermediate students and 11.2% for secondary students [35]. These findings are not supported by our study as we have found that more than 20% smoked both cigarettes and waterpipes. Nevertheless, if gender was to be taken into consideration, that study found that males and females shared equal percentages in their respective groups to smoking waterpipes only (26.3% vs. 25.0%), while males were superior to females in smoking both cigarettes and waterpipes (11.5% vs. 6.3%) [35]. 

In a study on smoking patterns conducted among schoolchildren in Lebanon, the average age of first-time experience with a cigarette was around 13 years old [36]. Furthermore, boys smoked more than girls, and the prevalence was increasing proportionally with the increase in age. Our results show that the smoking rates, daily consumption and starting age of the first experiment were significantly higher among males, those residing in the Beirut region, those who reported poor health perception and students with average-to-high FAS. A Lebanese study conducted in 2012 on grade 9 students revealed that the highest prevalence of cigarette smoking was found in Mount Lebanon (8.2%), followed by Beirut (4.9%) [36]. The findings of that study support ours that Beirut and Mount Lebanon areas have the highest prevalence of tobacco consumption. This could be explained by the more liberal attitudes and beliefs of people residing in these areas in comparison to the more traditional customs and habits of other Lebanese areas like Bekaa and the North of Lebanon. Tobacco smoking among Lebanese adolescents has also been attributed to many factors including peer pressure, socio-economic status and parents’ smoking habits. A study including 2443 students with an average age of 15 years found that the odds of smoking waterpipes were higher in older students of public schools and of lower socio-economic statuses. The rates of smoking also increased among those adolescents that had poor parental relationships and who had parents who were smokers [37]. A study has evaluated the socioeconomic differences in smoking in Jordan, Lebanon, Syria, and Palestine. It has revealed that wealth does not show a clear pattern and exerts a much weaker protective effect on cigarette smoking among men in Syria and Lebanon, and among Lebanese women only [38]. Moreover, men in the poorest wealth category in Palestine and women in the poor category in Syria have a lower risk of smoking than the richest [38]. The results of this study support our findings that a better socioeconomic status is not protective against smoking, but is associated with a higher proportion of tobacco use as we have reported. This could be attributed to the fact that those with better socioeconomic status have greater access to tobacco products, resulting in a higher consumption rate among this group.

Due to the COVID-19 pandemic, there was a delay in the publication of our data, which were collected during 2016 and 2017. There were no studies published in Lebanon on tobacco smoking and waterpipe use among adolescents during this period. However, we could not identify potential challenges to our study based on recent publications. According to the recent WHO Report on the Global Tobacco Epidemic [39], there were 1.07 billion smokers aged 15 years and above, worldwide. In addition, 24 million children aged 13–15 smoked globally [39]. Moreover, a systematic review on prevalence and trends in waterpipe tobacco smoking, in 2018, revealed that waterpipe tobacco smoking is most prevalent in Eastern Mediterranean and European countries, and appears higher among youth than adults [40]. 

### 4.1. Limitations and Strengths 

One of the main limitations in this study is the possibility of information bias that cannot be excluded in large-scale school-based studies due to self-reported data. Some students may not provide valid answers and may also underreport the level of smoking since the collected data are based on a self-administered questionnaire. This bias could have been minimized as the respondents were assured that their answers are anonymous, and that their parents and teachers would not see their answers. In addition, the importance of giving honest answers was highlighted during the administration of the questionnaires. Another limitation of this study may be related to selection bias. School dropouts were not included in the survey, this may have excluded a high-risk group for smoking. Moreover, information referring to smokeless tobacco is lacking in this survey. Finally, the nature of the cross-sectional study does not allow causal inferences to be made with any confidence. Despite these limitations, this study allows systematic monitoring of tobacco use among the youth, which is the first step in the planning of prevention strategies. The results of this study raise some important policy implications for the development of cigarette smoking prevention programs for the youth in Lebanon. 

### 4.2. Practical Implications

Our findings revealed a high cigarette and waterpipe experimentation among Lebanese adolescents. Such data can be relied on to interfere as policy makers, organisations, parents, health care providers, and schools to increase awareness and initiate interventions to protect our adolescents. 

## 5. Conclusions

The current study revealed an alarming rate of tobacco product use that constitutes a major public health issue for Lebanese adolescents that urgently needs intervention. The findings raise important policy implications for the development of cigarette smoking prevention programs for the youth. Future longitudinal studies to gain information about the determinants of cigarette smoking and waterpipe use among Lebanese adolescents are of extreme importance. 

## Figures and Tables

**Table 1:** Demographic characteristics of the participants.

	Beirut N = 751 (10.55%)	Mount Lebanon N = 4857 (68.25%)	North N = 473 (6.65%)	Bekaa N = 1036 (14.56%)	Total N = 1117
Age: Mean (±SD)	14.81 (±1.88)	15.46 (±1.91)	14.52 (±1.97)	14.85 (±2.28)	15.24 (± 2.00)
**Age**					
**11 years (%)**	1.3	0.4	1.6	5.4	1.3
**12 years (%)**	6.7	5.3	3.3	8.5	5.8
**13 years (%)**	20.6	10.4	33.6	24.1	15.0
**14 years (%)**	17.7	17.1	19.8	10.8	16.4
**15 years (%)**	20.2	18.8	19.2	9.4	17.6
**16 years (%)**	14.3	16.8	8.4	11.2	15.2
**≥17 years (%)**	18.9	30.9	13.7	30.4	28.4
**Gender**					
**Male (%)**	47.4	48.6	18.8	41.9	45.5
**Female (%)**	52.6	51.3	81.1	58.0	54.4
**FAS**					
**Low (%)**	21.1	27.6	59.8	25.5	28.8
**Medium (%)**	51.6	53.1	34.0	38.9	49.9
**High (%)**	27.3	19.3	6.2	35.6	21.3
**Excellent perceived health (%)**	36.5	43.1	38.3	53.1	43.5

**Table 2:** Cigarette smoking modalities according to gender, region, FAS and health perception.

Variable		Gender			Region					FAS				Health Perception		
		Male	Female	*p*-Value	Beirut	Mount Leb	North	Bekaa	*p*-Value	Low	Average	High	*p*-Value	Excellent	Not Excellent	*p*-Value
Smoking		N (%)			N (%)					N (%)				N (%)		
Smoking Experimentation	YES	1013 (31.9)	734 (19.1)	<0.001	237 (33.6)	1389 (28.6)	46 (10.0)	75 (7.6)	<0.001	426 (21.9)	869 (25.8)	380 (26.6)	0.002	589 (19.8)	1122 (29.1)	<0.001
Smoking Frequency	<1/w	192 (6.1)	142 (3.7)	<0.001	0 (0.0)	334 (6.9)	0 (0.0)	0 (0.0)	<0.001	90 (4.6)	180 (5.4)	58 (4.1)	<0.001	106 (3.6)	227 (5.9)	<0.001
	Min 1/w	164 (5.0)	107 (2.8)		32 (4.5)	206 (4.2)	9 (1.9)	18 (1.8)		61 (3.1)	118 (3.5)	77 (5.4)		87 (2.9)	174 (4.5)	
	1/day	229 (7.3)	117 (3.0)		116 (16.6)	185 (3.8)	18 (3.9)	27 (2.7)		77 (3.9)	148 (4.4)	87 (6.2)		102 (3.4)	226 (5.9)	
Packs per Day	<2	209 (9.0)	163 (4.2)	<0.001	57 (7.5)	362 (7.5)	15 (3.1)	18 (1.7)	<0.001	94 (4.8)	217 (6.5)	130 (9.2)	<0.001	137 (4.6)	307 (8.0)	<0.001
	>2	87 (2.7)	15 (0.3)		15 (2.0)	86 (1.7)	1 (0.2)	0 (0.0)		23 (1.1)	53 (1.5)	22 (1.5)		27 (0.9)	74 (1.9)	
Smoking Starting Age	≤11 y	169 (5.4)	63 (1.6)	<0.001	15 (2.2)	206 (4.2)	2 (0.4)	9 (0.8)	<0.001	49 (2.5)	119 (3.5)	58 (4.1)	0.004	80 (2.7)	150 (3.9)	<0.001
	12 y	126 (4.0)	87 (2.2)		23 (3.3)	179 (3.7)	4 (0.8)	7 (0.6)		52 (2.6)	97 (2.9)	64 (4.5)		77 (2.6)	135 (3.5)	
	13 y	118 (3.7)	88 (2.3)		23 (3.3)	174 (3.6)	2 (0.4)	7 (0.6)		43 (2.2)	111 (3.3)	46 (3.2)		63 (2.1)	141 (3.6)	
	14 y	129 (4.1)	88 (2.3)		24 (3.5)	187 (3.9)	3 (0.6)	3 (0.2)		56 (2.8)	112 (3.3)	44 (3.1)		70 (2.3)	143 (3.7)	
	15 y	99 (3.1)	90 (2.3)		18 (2.6)	168 (3.5)	1 (0.2)	2 (0.1)		56 (2.8)	100 (3.0)	29 (2.0)		62 (2.1)	127 (3.3)	
	≥ 16 y	106 (3.3)	91 (2.3)		25 (3.6)	164 (3.4)	6 (1.3)	2 (0.1)		57 (2.9)	98 (2.9)	38 (2.7)		57 (1.9)	139 (3.6)	

**Table 3:** Waterpipe consumption modalities according to gender, region, FAS and health perception.

Variable		Gender			Region					FAS				Health Perception		
		Male	Female	*p*-Value	Beirut	Mount Leb	North	Bekaa	*p*-Value	Low	Average	High	*p*-Value	Excellent	Not Excellent	*p*-Value
Water Pipe (WP)		N (%)			N (%)					N (%)				N (%)		
WP Experimentation	YES	1275 (40.3)	1144 (29.8)	<0.001	333 (47.6)	1920 (39.5)	83 (18.1)	83 (8.4)	<0.001	645 (33.30)	1207 (35.9)	482 (33.9)	0.113	856 (28.9)	1519 (39.4)	<0.001
WP Sessions per Week	<1	225 (7.7)	222 (5.8)	<0.001	33 (4.9)	388 (8.0)	11 (2.3)	15 (1.4)	<0.001	129 (6.68)	223 (6.7)	87 (6.2)	0.762	163 (5.5)	277 (7.2)	<0.001
	1–2	278 (8.8)	227 (5.9)		70 (10.5)	395 (8.2)	19 (4.1)	21 (2.0)		146 (7.56)	238 (7.1)	105 (7.5)		151 (5.1)	348 (9.1)	
	>2	216 (6.8)	186 (4.8)		87 (13.0)	275 (5.7)	28 (6.0)	12 (1.1)		108 (5.59)	184 (5.5)	94 (6.7)		129 (4.4)	263 (6.9)	
WP Starting Age	≤11 y	188 (6.0)	86 (2.2)	<0.001	23 (3.3)	233 (4.8)	4 (0.9)	14 (1.3)	<0.001	63 (3.27)	129 (3.8)	79 (5.6)	<0.001	100 (3.4)	172 (4.5)	<0.001
	12 y	181 (5.8)	157 (4.1)		40 (5.8)	291 (6.0)	4 (0.9)	3 (0.2)		66 (3.42)	189 (5.6)	78 (5.5)		125 (4.2)	213 (5.5)	
	13 y	174 (5.5)	165 (4.3)		49 (7.2)	276 (5.7)	6 (1.3)	8 (0.7)		74 (3.84)	190 (5.7)	69 (4.9)		112 (3.8)	224 (5.8)	
	14 y	207 (6.6)	223 (5.8)		47 (6.9)	367 (7.6)	10 (2.2)	6 (0.5)		130 (6.74)	224 (6.7)	64 (4.5)		137 (4.6)	285 (7.4)	
	15 y	148 (4.7)	182 (4.7)		35 (5.1)	280 (5.8)	9 (2.0)	6 (0.5)		111 (5.76)	152 (4.5)	63 (4.5)		101 (3.4)	227 (5.9)	
	≥16 y	133 (4.2)	182 (4.7)		44 (6.4)	253 (5.2)	17 (3.8)	1 (0.1)		122 (6.33)	149 (4.4)	39 (2.7)		111 (3.8)	202 (5.2)	

**Table 4:** Experimentation of both smoking and waterpipe among adolescents.

Variable		N (%)	*p*-Value
Region	Beirut	215 (30.8)	<0.001
	Mount Lebanon	1236 (25.4)	
	North	41 (9.0)	
	Bekaa	64 (6.5)	
	Total	1556 (22.2)	
Gender	Male	908 (28.7)	<0.001
	Female	648 (16.9)	
	Total	1556 (22.2)	
FAS	Low	382 (19.7)	0.002
	Average	774 (23.0)	
	High	336 (23.7)	
	Total	1492 (22.2)	
Perceived health	Excellent	527 (17.8)	<0.001
	Not Excellent	998 (25.9)	
	Total	1525 (22.4)	

## Data Availability

The data presented in this study are available on request from the corresponding author. The data are not publicly available because the data of this study is part of a larger dataset of national project.
